# Reply to Piewbang et al. Domestic Cat Hepadnavirus Antigens in Lymphoma Tissues. Comment on “Beatty et al. Domestic Cat Hepadnavirus and Lymphoma. *Viruses* 2023, *15*, 2294”

**DOI:** 10.3390/v16010149

**Published:** 2024-01-19

**Authors:** Julia A. Beatty, Thomas Tu, Patricia A. Pesavento, Joao P. Cavasin, Min-Chun Chen, Jonathan A. Lidbury, Joerg M. Steiner, Vanessa R. Barrs, John M. Cullen

**Affiliations:** 1 Department of Veterinary Clinical Sciences, Jockey Club College of Veterinary and Life Sciences, City University of Hong Kong, Hong Kong SAR, China; vanessa.barrs@cityu.edu.hk; 2 Centre for Animal Health and Welfare, City University of Hong Kong, Hong Kong SAR, China; 3 Storr Liver Centre, Westmead Clinical School and Westmead Institute for Medical Research, Faculty of Medicine and Health, The University of Sydney, Westmead, NSW 2145, Australia; t.tu@sydney.edu.au; 4 Sydney Infectious Diseases Institute, University of Sydney at Westmead Hospital, Westmead, NSW 2145, Australia; 5 Department of Pathology, Microbiology & Immunology, School of Veterinary Medicine, University of California, 4206 Vet Med 3A, Davis, CA 95616, USA; papesavento@ucdavis.edu; 6 Gastrointestinal Laboratory, College of Veterinary Medicine and Biomedical Sciences, Texas A&M University, College Station, TX 77843, USA; jcavasin@cvm.tamu.edu (J.P.C.); mcchen@cvm.tamu.edu (M.-C.C.); jlidbury@cvm.tamu.edu (J.A.L.); jsteiner@cvm.tamu.edu (J.M.S.); john_cullen@ncsu.edu (J.M.C.); 7 College of Veterinary Medicine, North Carolina State University, Raleigh, NC 27606, USA

We are grateful to the authors for providing additional data to demonstrate the presence of domestic cat hepadnavirus in lymphoma tissues [1], in response to our commentary [2]. It is unfortunate that *Veterinary Quarterly*, which published the original article [3], no longer accepts Letters to the Editor, even on appeal. We extend our sincere thanks to the Editor of *Viruses* for providing this platform for open scientific discussion.
